# CCR7A defines a subpopulation of IgD^+^IgM^-^ B cells with higher IgD secreting capacity in the rainbow trout skin

**DOI:** 10.3389/fimmu.2025.1538234

**Published:** 2025-02-14

**Authors:** Esther Morel, Juan German Herranz-Jusdado, Rocío Simón, Samuel Vicente-Gil, Lucía González, Carolina Tafalla

**Affiliations:** ^1^ Animal Biotechnology Department, National Institute of Agricultural and Food Research and Technology (INIA), Spanish Research Council (CSIC), Madrid, Spain; ^2^ Animal Health Research Center (CISA), National Institute of Agricultural and Food Research and Technology (INIA), Spanish Research Council (CSIC), Madrid, Spain; ^3^ Skretting Aquaculture Innovation, Stavanger, Norway

**Keywords:** IgD^+^IgM^-^ B cells, CCR7, skin, rainbow trout, plasma-like cells

## Abstract

B cells exclusively expressing IgD on the cell surface (IgD^+^IgM^−^ B cells) have been identified in mammals, where they seem to play a still not well-defined role in peripheral tolerance. These cells have also been reported in catfish (*Ictalurus punctatus*) peripheral blood and in several mucosal tissues of rainbow trout (*Oncorhynchus mykiss*), including gut, gills and skin. As in mammals, the precise function of these cells remains obscure, yet, in rainbow trout mucosal surfaces, these cells have been shown to be differentiated to plasma-like cells. Interestingly, in the gills, these IgD^+^IgM^−^ B cells expressed high levels of the CC chemokine receptor 7 (CCR7), receptor that in mammals controls the migration of B and T cells to secondary lymphoid organs. In this work, we have established that this is also true for the trout skin, where CCR7 defines a specific subset of IgD^+^IgM^−^ B cells that are further differentiated to a plasma-like profile than those not expressing CCR7. These findings increase the current understanding of this enigmatic B cell population and point to CCR7 as a key differentiation marker for these cells.

## Introduction

Despite the evolutionary conservation of immunoglobulin D (IgD), the specific functions of this Ig remain an enigma. In mammals, IgD is co-expressed on the surface of mature naïve B cells along with IgM. These surface IgM and IgD have identical antigenic specificities, since they are produced through the differential polyadenylation and alternative splicing of a heterogeneous pre-RNA encompassing the recombined V_H_DJ_H_ exon and the downstream Cμ and Cδ exons encoding the heavy chain constant (C) regions of IgM and IgD. Early studies undertaken with Cμ and Cδ mutants suggested both B cell receptors (BCRs) are interchangeable (1, 2), but alternative studies have pointed out some differences in their antigen recognition and downstream signaling, still not completely understood (3–5). In some cases, IgD can also be expressed through an alternative type of class-switch recombination (CSR), giving rise to B cells that exclusively express this Ig (6). These cells which are highly clonal (suggesting division) and have highly mutated Ig V_H_ regions have been shown to be often poly- and auto-reactive and therefore seem to play a relevant but not still well defined role in peripheral tolerance (7–9). Additionally, some of these IgD^+^IgM^−^ B cells differentiate to plasmablasts and plasma cells that secrete IgD. The function of secreted IgD in humans is largely unknown but a relevant mucosal role has been hypothesized based on recent evidence such as the fact that IgM-to-IgD CSR occurs mainly in nasopharyngeal and other upper respiratory tract compartments (10). Additionally, IgD has been shown to coat some respiratory pathogens (11), to establish an Fc receptor-independent interaction with innate populations such as basophils, mast cells, monocytes and myeloid dendritic cells (DCs) (12) and to be reactive to some food allergens (5, 13).

In teleost fish, naïve B cells also co-express IgM and IgD on the cell surface (14). As in mammals, these cells loose surface IgD as they engage on a differentiation program towards IgM-secreting plasmablasts or plasma-like cells (14). Nonetheless, IgD^+^IgM^-^ B cells are also present in teleost. These cells were first reported in catfish (*Ictalurus punctatus*) blood where they may account for up to 70% of peripheral B cells (15). Posterior studies described their presence in different mucosal surfaces of rainbow trout (*Oncorhynchus mykiss*) such as gills, intestine and skin, where they sometimes outnumber IgM-bearing populations (16–18). In the rainbow trout intestine, it has been established that this IgD^+^IgM^-^ population has the capacity to secrete an expanded and mildly mutated IgD that establishes a two-way interaction with the microbiota (17). However, as in mammals, many aspects of IgD regulation and functionality remain obscure.

In mammals, the CC chemokine receptor 7 (CCR7) controls the migration of immune cells to secondary lymphoid organs (19). In humans and mice, CCR7 has been shown to be expressed by different leukocyte types including dendritic cells (DCs), naïve B cells and different subsets of T cells including a subpopulation of memory cells designated as central memory T cells (19). These different T cell subpopulations home to the lymph nodes in response to CCR7 ligands (20). In the case of DCs, gene targeting has also shown that CCR7 is essential for the migration of DCs to draining lymph nodes, either in steady-state conditions or under inflammatory conditions, in tissues such as the skin (21, 22). In the case of B cells, it has been shown that follicular B cells down-regulate CXCR5 and up-regulate CCR7 upon antigen engagement (23).

A previous study from our group reported that in rainbow trout gills, the number of cells expressing CCR7 was much higher than in other tissues such as blood, thymus, spleen, kidney or gut (16). Interestingly, a large proportion of these CCR7^+^ cells in the gills were IgD^+^IgM^-^ B cells (16). Now, in the current study, we have focused on the rainbow trout skin, to establish that also in this tissue a large number of IgD^+^IgM^-^ B cells express this receptor on the cell surface. Interestingly, we have performed a series of experiments to compare the transcriptional profile and phenotype of IgD^+^IgM^-^CCR7^+^ and IgD^+^IgM^-^CCR7^-^ B cells, establishing that CCR7 defines a population of IgD^+^IgM^-^ B cells in the rainbow trout skin that are further differentiated towards a plasma-like profile. This study points to this chemokine receptor as a key differentiation maker for IgD plasma-like cells and contributes to a better understanding of this enigmatic B cell subset.

## Materials and methods

### Fish

Rainbow trout adults (15-20 cm) from a local fish farm (Piscifactoria Cifuentes, Guadalajara, Spain) were transferred to the animal facilities of the Animal Health Research Center (CISA-INIA-CSIC, Alalpardo-Valdeolmos, Spain). The animals were acclimatized for a period of 2 weeks before they were used. From that point, different fish were sacrificed to perform all the analysis described in this study. Fish were maintained at 14°C in recirculating water systems with external biofilters, continuous aeration, and under 12:12 h light-dark photoperiod. The animals were fed with a commercial diet (Skretting, Stavanger, Norway) twice a day.

### Tissue sampling and leukocyte isolation

Fish were euthanized by anesthetic overdose using a water bath with approximately 150 ppm of benzocaine (Sigma). Blood was extracted with a heparinized needle from the caudal vein and diluted 40 times with Leibovitz medium (L-15, Life Technologies) supplemented with 100 IU/ml penicillin, 100 μg/ml streptomycin (P/S), 10 units/ml heparin and 2% fetal calf serum (FCS) (all supplements also obtained from Life Technologies). Blood suspensions were layered onto 51% Percoll cushions and centrifuged at 400 x *g* for 30 min at 4°C, without brake. Blood leukocytes were collected from the interface and washed in L-15 containing P/S and 2% FCS.

Skin was sampled after extraction of blood to avoid blood leukocyte contamination. A piece of skin of approximately 10 cm^2^ was carefully collected from each side of the fish and placed on a petri dish with 2 ml of L-15 containing P/S and 2% FCS. There, all remaining muscle tissue was carefully removed, and after that, the skin was cut into small pieces and transferred to a tube containing L-15 with P/S and 5% FCS and 2 mg/ml dispase (Gibco). The skin fragments were incubated for 2 h at 4°C in continuous agitation. Subsequently, samples were pressed through a 100 µm nylon cell strainer (BD Biosciences). Samples were then washed by centrifugation (400 x *g* for 10 min) to remove cell debris. Clarified skin cell suspensions were layered onto 30/51% discontinuous Percoll (GE Healthcare) density gradients, and centrifuged at 400 x *g* for 30 min at 4°C, without brake. Cells at the interface, corresponding to skin leukocytes, were collected and washed in L-15 containing P/S and 5% FCS. Counting and cell viability were determined by trypan blue (Sigma) exclusion in all cases.

Other fish sacrificed and bled as described above were used to obtain samples of total skin, epidermis and dermis for total RNA isolation as described before (24). Finally, other fish were sacrificed to obtain skin samples for confocal microscopy.

### Flow cytometry

Isolated blood and skin leukocytes were washed with staining buffer (phenol red-free L-15 medium supplemented with 2% FCS and P/S) and incubated for 1 h at 4°C with 2 µg/ml of a specific anti-trout CCR7 polyclonal antibody (pAb) obtained in rabbit and previously described (16), in staining buffer. After this time, cells were washed with staining buffer and stained with a secondary Ab Alexa Fluor 488 goat anti-rabbit IgG (4 µg/ml) (Innova Biosciences) for 30 minutes at 4°C in darkness. Cells were washed again as described above and stained with anti-trout IgD [mAb mouse IgG1 coupled to allophycocyanin (APC); 5 µg/ml] (25) and anti-trout IgM [1.14 mAb mouse IgG1 coupled to R-phycoerythrin (R-PE); 1 µg/ml] (26), diluted in staining buffer, for 1 h at 4°C in darkness. All antibodies were fluorescently labeled using R-PE or APC Lightning-Link labeling kits (Innova Biosciences) following the manufacturer’s instructions. Finally, cells were washed twice in staining buffer and analyzed in a FACS Celesta flow cytometer (BD Biosciences) equipped with a BD FACSDiva software (BD Biosciences). Data from the flow cytometer were analyzed using the software FlowJo^®^ v.10 (FlowJo LLC, Tree Star). In all cases, cell viability was checked using 4’,6-diamine-2’-phenylindole dihydrochloride (DAPI) at 0.2 µg/ml.

### Cell sorting

Blood and skin leukocytes isolated and stained as described above were used to sort IgM^+^IgD^-^, IgD^+^IgM^-^, IgD^+^IgM^-^CCR7^-^ and IgD^+^IgM^-^CCR7^+^ B cells from skin, and IgM^+^IgD^+^ B cells from blood, on a FACSAria™ III flow cytometer (BD Biosciences) equipped with BD FACSDiva™ software. The cell sorting was performed on the basis of the fluorescence from the anti-trout CCR7 (Alexa Fluor 488), the anti-trout IgM coupled to R-PE and/or the anti-trout IgD coupled to APC, after staining the cells with the specific antibodies as described above. Approximately 10,000 cells from each subset were collected in staining buffer for subsequent RNA isolation. An equal number of IgD^+^IgM^-^CCR7^-^ and IgD^+^IgM^-^CCR7^+^ B cells B cells were also sorted from each fish to compare the IgD-secreting capacity of these B cell subsets by ELISA.

To undertake the confocal analysis of IgD^+^IgM^-^CCR7^-^ and IgD^+^IgM^-^CCR7^+^ B cells from the skin, isolated skin leukocytes not stained with antibodies were cell sorted exclusively on the basis of FSC and SSC parameters to obtain an enrichment of skin lymphoid-like cells (small cells with low complexity).

In all cases, cell viability was checked using 7-Aminoactinomycin D (7-AAD) (BD Biosciences) at a 1:100 dilution.

### Confocal microscopy

Sorted skin lymphocytes were seeded on a poly-L-lysine (0.01% solution)-coated slide and incubated at room temperature (RT) for 1 h in a humidified chamber. Thereafter, the samples were fixed in 4% paraformaldehyde solution for 30 min at RT and incubated for 1 h with blocking solution (TBS with 5% BSA and 0.5% saponin). Samples were then incubated with the anti-trout CCR7 (20 µg/ml) in combination with anti-trout IgM coupled to APC (2 μg/ml) or anti-trout IgD coupled to APC (50 μg/ml) for 1 h at RT in a humidified chamber. After that, cells were washed with PBS 1x and were subsequently stained with a secondary Ab Alexa Fluor 488 goat anti-rabbit IgG (20 µg/ml) for 1 h at RT. Samples incubated with secondary Alexa Fluor 488 goat anti-rabbit IgG along with APC-conjugated mouse IgG1 isotypes at the same concentrations were also included to confirm the specificity of the antibody signals. Slides were counterstained with 1 μg/ml of DAPI (Sigma-Aldrich) for 10 min, rinsed with PBS 1x and mounted with Fluoromount (Sigma-Aldrich).

For the confocal microscopy analysis of the skin, skin tissue pieces obtained as described above were fixed in 4% paraformaldehyde and processed for paraffin embedding following routine histological procedures. Thereafter, 4 μm-thick tissue sections were mounted on Superfrost Plus slides (Thermo Scientific), and a double immunofluorescent detection of IgD and CCR7 performed. For this, antigen retrieval was performed by heating in Tris–EDTA buffer (10 mM Tris base, 1 mM EDTA, pH 9) in a microwave oven for 5 min at 800 W and 5 min at 450 W. Thereafter, non-specific binding was blocked with 5% bovine serum albumin (BSA) in Tris-buffered saline (TBS). Tissues were then incubated with the anti-trout-IgD (15 μg/ml) and anti-trout CCR7 (10 μg/ml) Abs. Thereafter, tissue sections were rinsed with TBT and TBS 1x followed by an incubation with a secondary anti-mouse IgG antibody conjugated with AlexaFluor^®^647 (ThermoFisher) (20 μg/ml) in combination with an anti-rabbit IgG AlexaFluor^®^488 (ThermoFisher) (20 μg/ml). Sections were then counterstained with DAPI (1 μg/ml, Sigma). All the incubation steps were performed for 1 h at RT in the dark. Tissue autofluorescence was blocked by incubation with 0.3% Sudan black B (Sigma-Aldrich) in 70% ethanol for 10 min. Sections were then rinsed with TBS and mounted with Fluoromount.

All images were obtained using a laser scanning confocal microscope (Zeiss Axiovert LSM 880) and they were further processed with Adobe Photoshop CS6 software.

### Real-time PCR analysis

Total RNA was isolated from sorted skin IgM^+^IgD^-^ and IgD^+^IgM^-^ B cells, sorted blood IgM^+^IgD^+^ B cells, and sorted skin IgD^+^IgM^-^CCR7^+^ and IgD^+^IgM^-^CCR7^-^ B cells using the Power SYBR Green Cells-to-Ct Kit (Invitrogen) following manufacturer’s instructions. RNA was treated with DNase I (Thermo Fisher Scientific) during the process to remove genomic DNA that might interfere with the reactions. Reverse transcription was also performed using the Power SYBR Green Cells-to-Ct Kit following the manufacturer’s instructions. To evaluate the levels of transcription of *ccr7a, ccr7b*, *mhc ii*, *il1b, prdm1a-1*, *prdm1a-2*, *prdm1c-1*, *prdm1c-2*, *bcma*, *irf4*, *pax5*, and secreted *igd* genes, real-time PCR was performed with a LightCycler^®^ 96 System instrument (Roche) using SYBR Green PCR core Reagents (Applied Biosystems) and specific primers previously described (**Supplementary Table 1**). Each sample was measured under the following conditions: 10 min at 95°C, followed by 45 amplification cycles (15 s at 95°C and 1 min at 60°C). A dissociation curve was obtained by reading fluorescence every degree between 60°C and 95°C to ensure only a single product had been amplified. The expression of individual genes was normalized to the relative expression of *b-actin*, that was selected as a housekeeping gene following the MIQE guidelines (27). The expression levels were calculated using the 2^−ΔCt^ method, where ΔCt is determined by subtracting the *b-actin* value from the target Ct. Negative controls with no template and *minus*-reverse transcriptase (-RT) controls were included in all experiments.

Total skin, epidermis and dermis samples were used to isolate total RNA using TRI Reagent Solution (Thermo Fisher Scientific, USA) following the manufacturer’s instructions. RNA pellets were washed with 75% ethanol, dissolved in RNAse-free water and stored at -80°C until use. One μg of RNA was treated with DNAse and then used to obtain cDNA using the Superscript II reverse transcriptase (Thermo Fisher Scientific) and oligo(dT)_23_VN, following the manufacturer´s instructions. The cDNA was diluted in a 1:10 proportion with RNAse-free water and stored at -20°C until use. In these samples, we evaluated the levels of transcription of *ccr7a, ccr7b*, and *igd* by real-time PCR using a LightCycler96 System, FastStart Essential DNA Green Master reagents (Roche) and specific primers (**Supplementary Table 1**). Each sample was measured under the following conditions: 10 min at 95°C, followed by 40 amplification cycles (10 s at 95°C, 10 s at 60°C and 10 s at 72°C). A dissociation curve was obtained by reading fluorescence every degree between 72°C and 95°C to ensure only a single product had been amplified. The expression of individual genes was normalized to the relative expression of *b-actin* as described above.

### Analysis of IgD secretion by ELISA

Sorted skin IgD^+^IgM^-^CCR7^-^ and IgD^+^IgM^-^CCR7^+^ leukocytes were collected in L-15 medium supplemented with P/S and 10% FCS, and cultured for 48 h at 20°C. After this time, supernatants were collected to further evaluate the levels of secreted IgD by ELISA. For this, 96 well-ELISA plates were coated overnight at 4°C with 100 μl of a mixture of six different anti-light chain (IgL) antibodies (2A1, 2H9, 2D12, 1B4, 3E4 and 1A6) (28) diluted in 0.05 M carbonate buffer pH 9.7 at a final concentration of 5 μg/ml. This protocol was optimized using antibodies against the IgL because the detection of monomeric proteins such as IgD is more efficient when different antibodies are used in the coating and the detection steps. Following three washing steps in PBS containing 0.05% Tween-20 (PBS-T), non-specific binding sites were blocked with 100 μl of 5% BSA in PBS for 1 h at RT. Thereafter, plates were washed again three times with PBS-T. Cell supernatants diluted 1:2 in PBS supplemented with 1% BSA were then added to the wells and incubated for 1 h at RT. After three washing steps with PBS-T, 100 μl of biotinylated anti-trout IgD mAb (1 μg/ml) diluted in 1% BSA in PBS were added to the wells and then incubated for 1 h at RT. The wells were washed three times again and wells incubated with 100 μl of Streptavidin-HRP (diluted in 1:1000 in 1% BSA in PBS) for 1 h at RT. After another 3 washes, 100 μl of OPD (O-phenylenediamine Dihydrochloride) (Sigma) were added (1 mg/ml). The reaction was stopped by adding 50 μl of 2.5 M H_2_SO_4_ and absorbance at 490 nm was measured in a FLUO Star Omega Microplate Reader (BMG Labtech). Positive and negative controls were included in all the plates.

### Statistical analysis

All data were analyzed using GraphPad Software (GraphPad Prism v8.0.1, La Jolla California, USA). Data were checked for normality using the Shapiro-Wilk test and q-q plots. A two-tailed Student´s *t*-test was used for normally distributed data, whereas non-normally distributed data were tested with a non-parametric Wilcoxon matched-pairs signed-rank test. The differences between the mean values were considered significant on different degrees, where * means *p* ≤ 0.05, ** means *p* ≤ 0.01 and *** means *p* ≤ 0.001. Numbers of individual fish used in each experiment (biological replicates) are detailed in figure legends (*n*).

## Results

### CCR7 is preferentially expressed in IgD^+^IgM^-^ B cells in the trout skin

**Figure 1:**
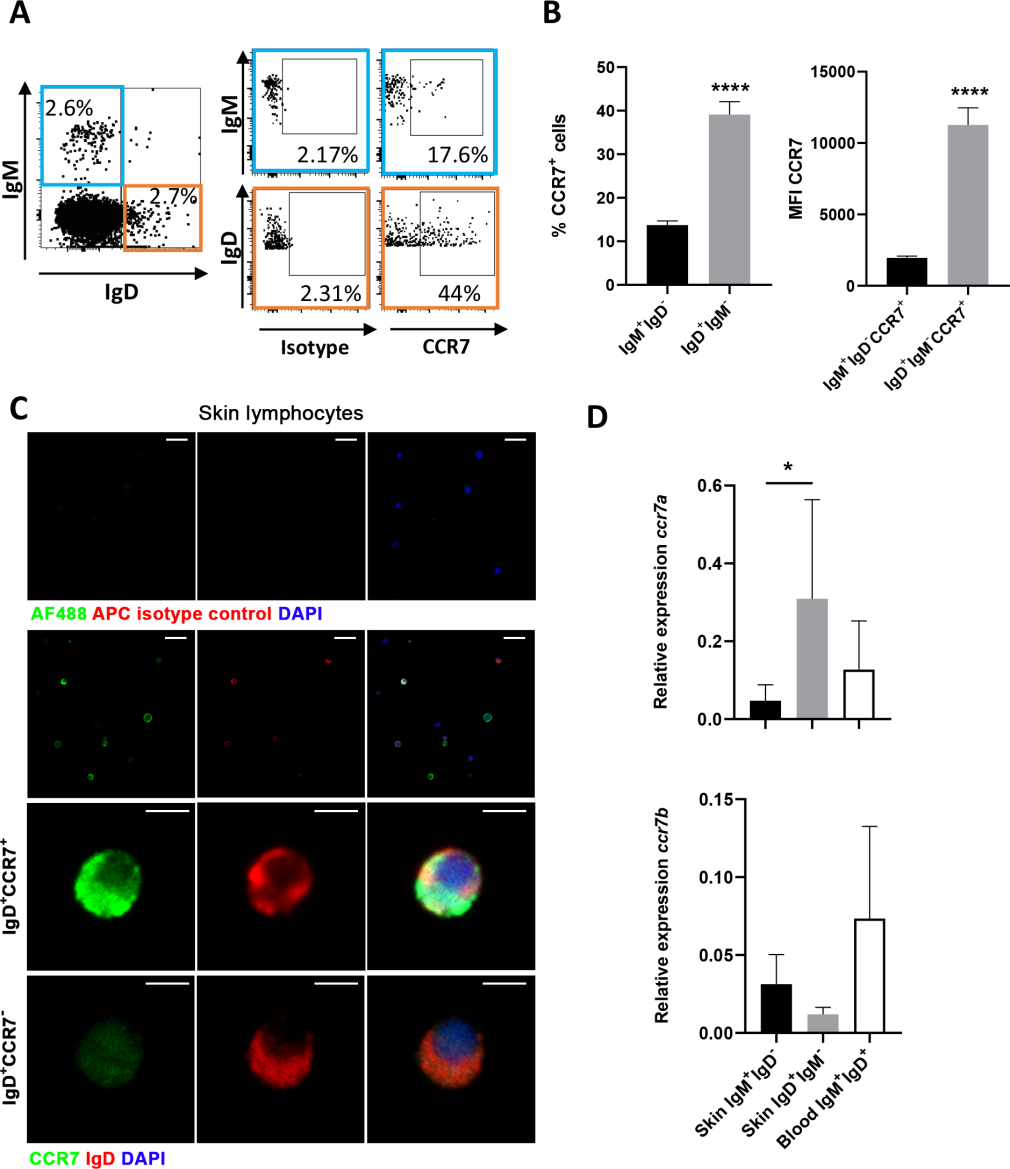
CCR7 is preferentially expressed in IgD^+^IgM^-^ B cells in the trout skin. Skin leukocytes were isolated and stained with anti-trout IgM and anti-trout IgD specific mAbs and with a polyclonal anti-trout CCR7 Ab. **(A)** Dot plots show the percentages of skin IgM^+^IgD^-^CCR7^+^ and IgD^+^IgM^-^CCR7^+^ B cells in a representative fish. **(B)** Graphs show the mean percentages of CCR7^+^ cells in skin IgM^+^IgD^-^ and IgD^+^IgM^-^ cells and the mean fluorescence intensity (MFI) values of CCR7 on skin IgM^+^IgD^-^CCR7^+^ and IgD^+^IgM^-^CCR7^+^ B subsets (mean + SEM, n = 20 independent fish). **(C)** Skin leukocytes were sorted according to FSC (size) and SSC (complexity) parameters to select lymphoid cells (small cells with low complexity) for immunofluorescence analysis under the confocal microscope. Images of isotype controls are shown in the upper panel. Visualization of skin IgD^+^IgM^-^CCR7^+^ and IgD^+^IgM^-^CCR7^-^ B cells are shown in the lower panels, with examples of each subset below. Scale bars: 20 µm (first and second rows); 5 µm (third and fourth rows). **(D)** Skin IgM^+^IgD^-^ and IgD^+^IgM^-^ B cells and blood IgM^+^IgD^+^ B cells were sorted to determine the mRNA levels of *ccr7a* and *ccr7b* by real-time PCR. Graphs show the relative expression of *ccr7a* and *cr7b* in these three subpopulations (mean + SEM, n = 4-6 independent fish). Statistical differences were evaluated by a paired two-tailed Student’s *t*-test when data were normally distributed, whereas non-normally distributed data were analyzed with the non-parametric Wilcoxon matched-pairs signed-rank test. Asterisks denote significantly different values among subpopulations as indicated (*p ≤ 0.05; ****p ≤ 0.001).

As previously reported (18), within the IgM/D lineage, most of the cells identified in the rainbow trout skin correspond to cells that only express one Ig, either IgM (IgM^+^IgD^-^ B cells) or IgD (IgD^+^IgM^-^ B cells). When we analyzed the levels of surface CCR7 expression in these two populations, by means of a specific antibody previously characterized (16), using the gating parameters indicated in **Supplementary Figure 1**, we observed that while the percentage of IgM^+^IgD^-^ B cells expressing CCR7 was low (~11%), the percentage of IgD^+^IgM^-^CCR7^+^ B cells found in skin was ~39% (**Figures 1A, B**). Furthermore, within the populations expressing CCR7, the levels of surface CCR7 expression (CCR7 mean fluorescence intensity, MFI) were significantly higher (~11000) on skin IgD^+^IgM^-^ B cells than on skin IgM^+^IgD^-^ B cells (~1900) (**Figures 1A, B**). The existence of IgD^+^IgM^-^ B cells expressing or not the chemokine receptor was confirmed by confocal microscopy (**Figure 1C**), after sorting skin lymphocytes according to FSC and SSC parameters (small cells with low complexity). As shown in the image, both IgD^+^IgM^-^CCR7^+^ and IgD^+^IgM^-^CCR7^-^ B cells were identified within skin leukocyte populations. Albeit at a lower frequency, IgM^+^IgD^-^CCR7^+^ cells were also identified in the cultures (**Supplementary Figure 2**), together with a majority of IgM^+^IgD^-^CCR7^-^ B cells.

Interestingly, rainbow trout are known to contain two homologue genes for mammalian *ccr7*, designated as *ccr7a* and *ccr7b*. Because the anti-CCR7 antibody used in this study recognizes both proteins (16), to get an indication of whether one of these genes could be preferentially expressed in skin IgD^+^IgM^-^ B cells, we sorted skin IgM^+^IgD^-^ and IgD^+^IgM^-^ B cells and blood IgM^+^IgD^+^ B cells for comparison, and analyzed the levels of transcription of the two isoforms by real-time PCR. As shown in **Figure 1D**, skin IgD^+^IgM^-^ B cells present significantly higher mRNA levels of *ccr7a* than skin IgM^+^IgD^-^ cells. These levels were also higher than those detected in blood IgM^+^IgD^+^ B cells, but in this case, the differences were not significant (**Figure 1D**). On the other hand, the levels of transcription of *ccr7b* were not significantly different among the B cell populations studied, with slightly higher levels in blood IgM^+^IgD^+^ B cells than in the skin B cell populations (**Figure 1D**).

### Distribution of IgD^+^IgM^-^CCR7^+^ B cells in the trout skin

**Figure 2:**
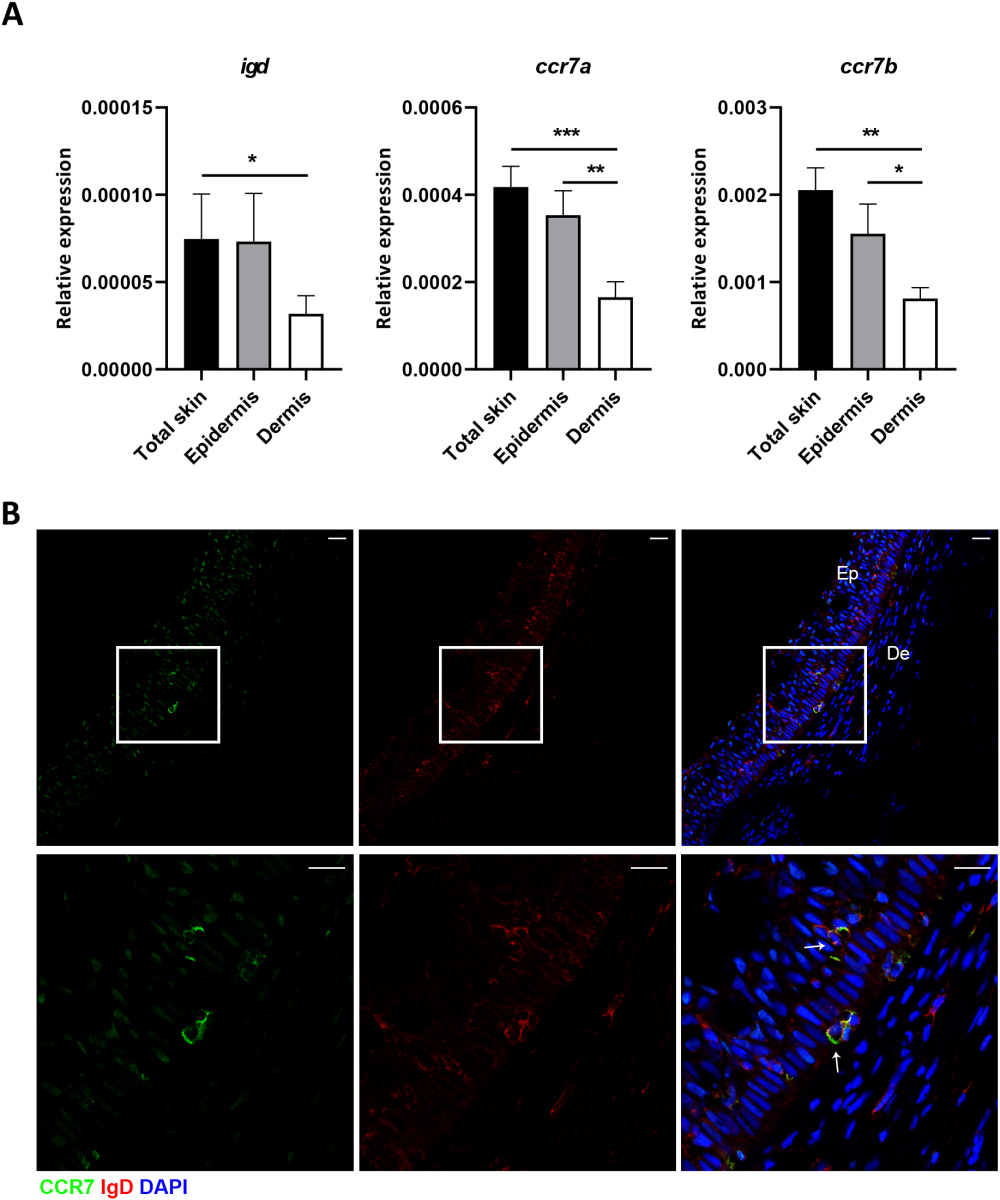
IgD^+^IgM^-^CCR7^+^ B cells are localized in the epidermis. **(A)** Real-time PCR analysis of *igd, ccr7a* and *ccr7b* in total skin, epidermis and dermis samples. Graphs show levels of transcription of *igd, ccr7a* and *ccr7b* in the different samples. Results are shown as the mean gene expression relative to the expression of an endogenous control (*b-actin*) + SEM (n = 7 independent fish). Statistical differences were evaluated by a paired two-tailed Student’s *t*-test, and asterisks denote significant differences among different B cell subsets as indicated (*p ≤ 0.05; **p ≤ 0.01; ***p ≤ 0.001). **(B)** Confocal microscopy images from skin in a representative fish showing IgD and CCR7 staining in the dermis and epidermis. Scale bars: 20 µm (upper panels); 5 µm (lower panels).

We next decided to investigate whether there was a specific localization of this IgD^+^IgM^-^CCR7^+^ B cell subset in trout skin. As a first step, we analyzed the expression of *igd, ccr7a* and *ccr7b* genes by real-time PCR in total skin samples as well as in samples exclusively containing epidermis or dermis, obtained as previously described (24). As shown in **Figure 2A**, the levels of transcription of *igd, ccr7a* and *ccr7b* were significantly higher in total skin samples than in samples exclusively containing dermis. In the case of *ccr7a* and *ccr7b*, the mRNA levels in epidermis samples were also significantly higher than those detected in dermis samples (**Figure 2A**). These results suggested that IgD^+^ B cells, especially those expressing CCR7, were located in the epidermis layer. This was confirmed through confocal microscopy. As shown in **Figure 2B**, although IgD was detected both in the epidermis and the dermis layers, CCR7 expression seemed clearly restricted to the epidermis layer (**Figure 2B**). Consequently, IgD^+^IgM^-^ B cells expressing CCR7 receptor were exclusively visualized in the epidermis (**Figure 2B**).

### Transcriptional profile of skin IgD^+^IgM^-^ B cells expressing CCR7 or not

**Figure 3:**
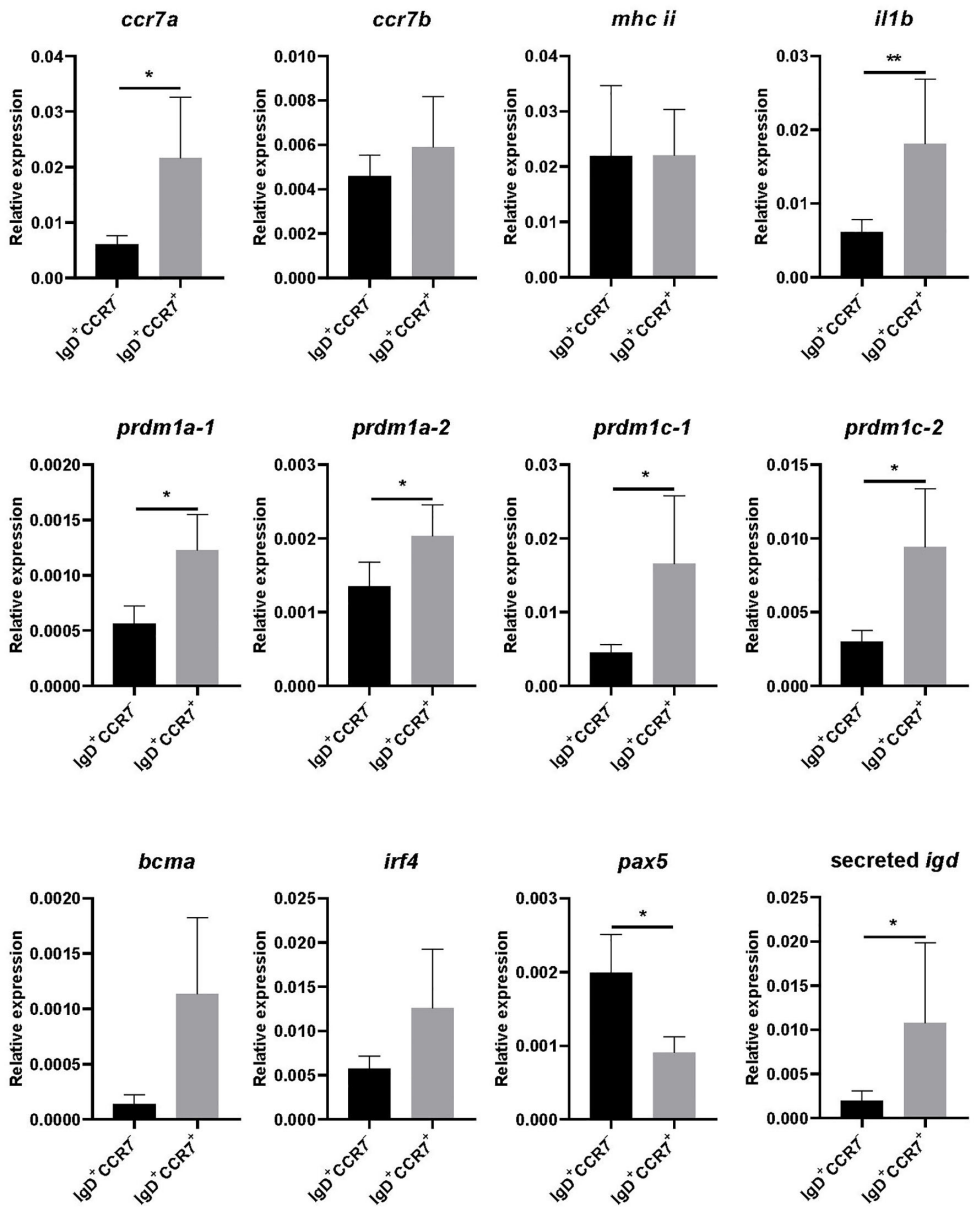
Transcriptional profile of skin IgD^+^IgM^-^CCR7^+^ and IgD^+^IgM^-^CCR7^-^ B cells. Skin leukocytes were isolated and stained with anti-trout IgM and anti-trout IgD specific mAbs and with a polyclonal anti-trout CCR7 Ab. Thereafter, IgD^+^IgM^-^ B cells expressing or not CCR7 were FACS-sorted to determine the levels of transcription of several genes by real-time PCR. Graphs show levels of transcription of *ccr7a, ccr7b*, *mhc ii*, *il1b*, *prdm1a-1*, *prdm1a-2*, *prdm1c-1*, *prdm1c-2*, *bcma*, *irf4*, *pax5* and secreted *igd*, in each B cell subset. Results are shown as the mean gene expression relative to the expression of an endogenous control (*b-actin*) (mean + SEM, n = 6-10 independent fish). Statistical differences were evaluated by a paired two-tailed Student’s *t*-test when data were normally distributed, whereas non-normally distributed data were analyzed with the non-parametric Wilcoxon matched-pairs signed-rank test. Asterisks denote significant differences between different B cell subsets as indicated (*p ≤ 0.05; **p ≤ 0.01).

We next compared the transcriptional profile of skin IgD^+^IgM^-^CCR7^+^ B cells to that of skin IgD^+^IgM^-^CCR7^-^ B cells after isolating both subpopulations by cell sorting. We first evaluated the levels of *ccr7a* and *ccr7b* transcription and observed that while *ccr7a* mRNA levels were significantly higher in IgD^+^IgM^-^CCR7^+^ B cells compared to IgD^+^IgM^-^CCR7^-^ B cells, there were no differences in *ccr7b* mRNA levels in these two subpopulations (**Figure 3**).

To further characterize the two B cell subsets, we also studied the levels of transcription of *mhc ii*, *il1b*, four genes that code for Blimp1 homologues in rainbow trout (*prdm1a-1*, *prdm1a-2, prdm1c-1* and *prdm1c-2)* (29), *bcma*, *irf4*, *pax5* and secreted *igd*. Our results show that IgD^+^IgM^-^CCR7^+^ B cells transcribe significantly higher levels of *il1b* (**Figure 3**). Additionally, they also transcribed higher levels of genes related to B cell differentiation such as *prdm1a-1*, *prdm1a-2, prdm1c-1* and *prdm1c-2* (**Figure 3**). Also in correlation with a more differentiated profile of cells expressing CCR7, we detected lower *pax5* and higher secreted *igd* transcription levels in IgD^+^IgM^-^CCR7^+^ B cells than in IgD^+^IgM^-^CCR7^-^ B cells (**Figure 3**).

### Skin IgD^+^IgM^-^ cells expressing CCR7 have a phenotype of cells that are further differentiated towards a plasma-like cell profile

**Figure 4:**
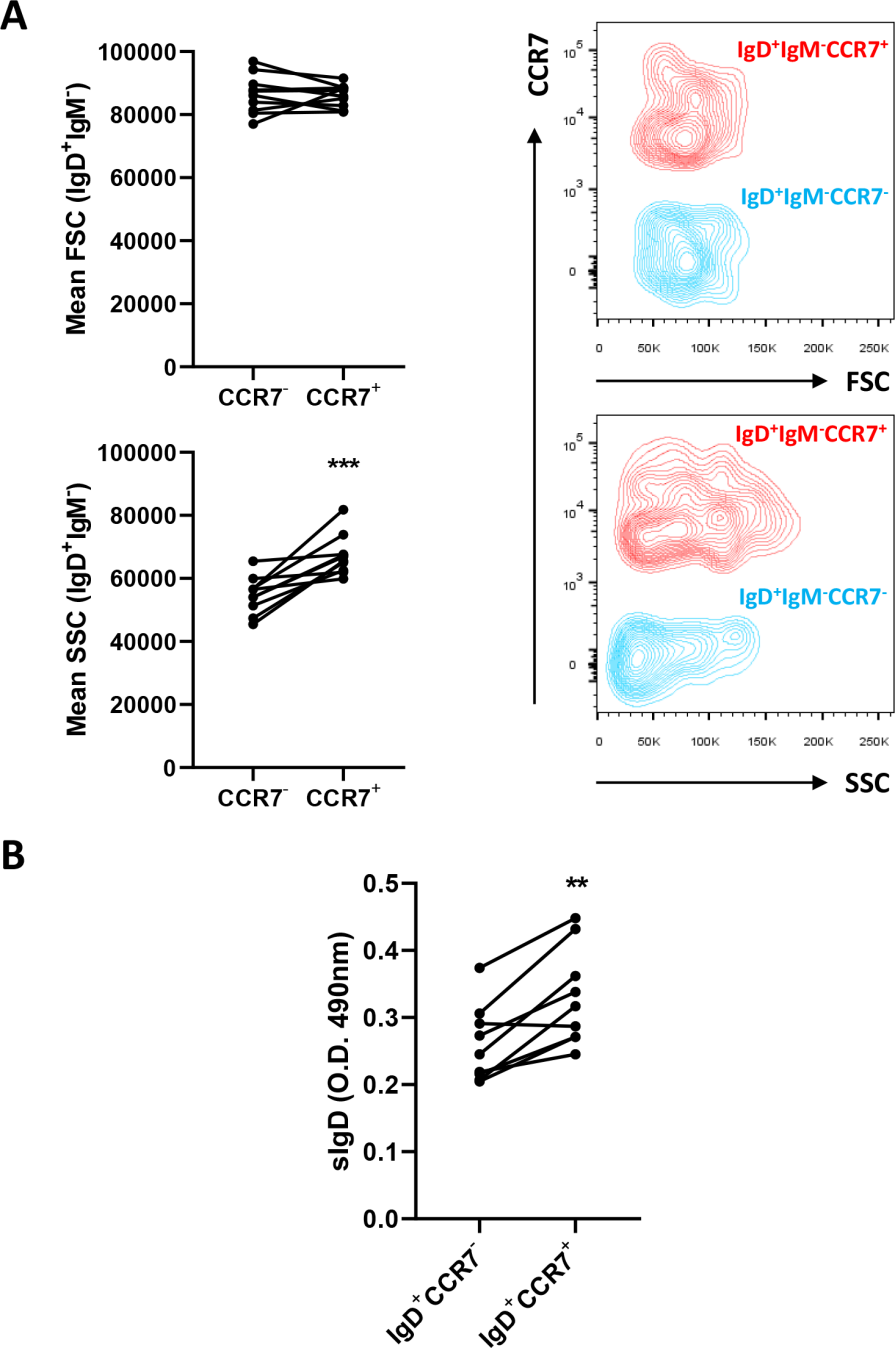
IgD^+^IgM^-^CCR7^+^ B cells are more granular and secrete more IgD than IgD^+^IgM^-^CCR7^-^ B cells. Skin leukocytes were isolated and stained with anti-trout IgM and anti-trout IgD specific mAbs and with a polyclonal anti-trout CCR7 Ab. **(A)** Comparison of the size (FSC values) and internal complexity (SSC values) by flow cytometry in IgD^+^IgM^-^CCR7^+^ and IgD^+^IgM^-^CCR7^-^ B cell subsets. Representative dot plots are included along with graphs showing mean fluorescence intensity (MFI) values of FSC and SSC for each fish (n = 10 independent fish) in both B cell subsets. **(B)** Skin IgD^+^IgM^-^CCR7^+^ and IgD^+^IgM^-^CCR7^-^ B cells were FACS-sorted and plated for 48 h at 20°C. After that time, culture supernatants were collected and the amount of secreted IgD estimated by ELISA. Graph shows the absorbance values at 490 nm obtained in supernatants from different sorted subpopulations (n = 9 independent fish). Statistical differences were evaluated by a paired two-tailed Student’s *t*-test, and asterisks denote significant different values between subpopulations as indicated (**p ≤ 0.01; ***p ≤ 0.001).

The transcriptional profile of IgD^+^IgM^-^CCR7^+^ and IgD^+^IgM^-^CCR7^-^ B cells pointed to cells expressing CCR7 as being further differentiated towards a plasma-like profile. Because B cell differentiation can affect the size and complexity of the cells, we analyzed both the size (referred to as forward scatter, FSC) and granularity or internal complexity (referred to as side-scattered, SSC) of these cell types by flow cytometry. As shown in **Figure 4A**, IgD^+^IgM^-^CCR7^+^ B cells were found to be significantly more granular than IgD^+^IgM^-^CCR7^-^ B cells. However, CCR7 expression did not seem to influence the size of IgD^+^IgM^-^ B cells, as no significant differences were observed between these two groups when this parameter was studied (**Figure 4A**).

Furthermore, we also sorted the two IgD^+^IgM^-^ B cell populations, cultured them for 48 h and analyzed their capacity to secrete IgD into the culture supernatants. As indicated in **Figure 4B**, this experiment revealed that the levels of secreted IgD produced by IgD^+^IgM^-^CCR7^+^ B cells were significantly higher than those of IgD^+^IgM^-^CCR7^-^ B cells.

## Discussion

Chemokines are cytokines that regulate the migration and localization of leukocytes in homeostasis as well as their recruitment to sites of inflammation and infection (30). These chemokines attract and modulate the immune function of the recruited cells through the interaction with specific G protein-linked chemokine receptors that form a family of structurally and functionally related proteins (31). Chemokines are defined by the presence of four conserved cysteine residues and are divided into four subfamilies based on the distinctive pattern of the two N terminal cysteines: CXC, CC, C and CX_3_C classes (32). In mammals, the CC chemokines CCL19 and CCL21 are known to signal through CCR7 (19). In fish, although homologues of CCR7 have been identified in different teleost fish species (two in rainbow trout) (33), the ligands for CCR7 have not yet been defined, as the expansion of chemokine genes in most teleost fish species has rendered the adscription of true homologues a difficult task (32).

In mammals, CCR7-mediated signals control the migration of immune cells to secondary lymphoid organs (19). In the current work, we have identified that the rainbow trout skin constitutes an important site for CCR7^+^ cells. Among these cells, a high proportion were IgD^+^IgM^-^ B cells (14.6% ± 8%) and a few IgM^+^IgD^-^ B cells (2.7% ± 2.2%). Of course, the rest of the CCR7^+^ cells identified in the skin may correspond to other leukocyte subsets, possibly T cells or DCs, although in trout gills, CD8^+^ T cells do not express CCR7 on the cell membrane (16). Nonetheless, the focus of this study was to examine CCR7 expression specifically on B cell subsets, therefore what other leukocyte subsets express this chemokine receptor will be the focus of future studies as more tools to differentiate leukocytes become available in fish.

As mentioned before, mammalian follicular B cells, upon activation, experience a change in the pattern of expression of chemokine receptors. These changes involve the up-regulation of CCR7 expression which renders B cells more responsive to chemokines produced in the T cell zone such as CCL19 and CCL21, thereby promoting their migration to T cell areas (23). In rainbow trout skin, only a small percentage of IgM^+^IgD^-^ B cells expressed CCR7, and even in this case, the levels of expression were much lower than those of IgD^+^IgM^-^CCR7^+^ B cells. It has to be taken into account that teleost fish do not have lymph nodes and the spleen constitutes the main secondary lymphoid tissue (34). Additionally, although lymphoid microstructures that carry out some of the functions of germinal centers (GCs) have been recently described in the rainbow trout spleen (35), teleost fish do not organize true GCs. Hence, teleost B cell responses are mainly extrafollicular, and therefore the changes that fish IgM^+^ B cells undergo during differentiation might not be exactly the same as those of mammalian follicular B cells. No organized structures are present in mucosal surfaces either, where B and T cells are scattered in a seemingly disorganized fashion (36). Because of all this, the degree of T cell help that fish B cells receive to differentiate, if any, is still unknown, especially in mucosal surfaces, being this a factor that might also condition the expression pattern and the role of CCR7 in fish.

Teleost fish exclusively express three Ig isotypes, namely IgM, IgD and IgT. IgT is a teleost-specific Ig, present in most, but not all, teleost fish species (37). Because IgT generates antigen recognition diversity through a private VDJ gene cassette, IgT production is completely independent to that of IgM and IgD (37), and therefore, IgT-expressing cells constitute an independent lineage of B cells from that of IgM/D subsets (38). IgT responses were shown to be dominant in different mucosal compartments after exposure to specific infections (38–40), which led to the hypothesis that IgT was a mucosally-dedicated Ig in fish, in the absence of IgA. However, it seems probable, that this is not a general hypothesis, since posterior studies have revealed IgT responses outside mucosal compartments (41, 42) and also mucosal Ig responses not involving IgT (43, 44). Based on this and other recent evidence (45), it seems obvious that the involvement of different B cell subsets in fish mucosal responses is dependent on many factors including the fish species; the temperature; the mucosal tissue examined; or the type of pathogen or stimulation, among others. Additionally, it seems probable that there is a collaborative response of different B cell subsets, including cells bearing all three Ig isotypes, as a relevant mucosal role of IgD in teleost fish seems now evident (17, 18, 46).

Although B cells co-expressing both IgM and IgD constitute the main B cell subset in systemic compartments in teleosts, in mucosal tissues such as gills or skin, most of the cells identified within the IgM/D lineage correspond to cells that exclusively express one or the other Ig (18). Interestingly, in non-immunized and apparently healthy individuals, these IgM^+^IgD^-^ and IgD^+^IgM^-^ B cells were already differentiated to a plasma-like profile and were able to secrete the corresponding Ig (18). Yet, upon bacterial infection, IgM^+^IgD^-^ B cells were capable of differentiating further towards plasma-like cells, since they increased in size, up-regulated the transcription of differentiation markers and secreted specific IgM (45). In that experiment, *Yersinia ruckeri* did not exert a visible effect on IgD^+^IgM^-^ populations. However, to the light of the results reported in the current study, it seems that although IgD^+^IgM^-^ B cells always correspond to cells that have already started a differentiation program towards plasmablasts/plasma-like cells, these cells can also be found in different differentiation states, with CCR7 as a marker for the cells that have differentiated further. Whether IgD^+^IgM^-^ B cells expressing CCR7 correspond to cells that have engaged a specific antigen or whether these two types of cells co-exist in the mucosa in homeostasis remains unknown. Nonetheless, it has to be taken into account that the fish used to conduct all these studies come from a fish farm at adult age and therefore have been exposed to a plethora of different microbes before they were sampled.

Although the transcriptional profile of IgD^+^IgM^-^ B cells was already that of a cell that had started a differentiation program towards plasmablasts/plasma-like cells (18), when we compared that of cells that specifically expressed CCR7 to those that do not, we observed that the CCR7^+^ subsets transcribed at higher levels the four homologues of *prdm1a* found in rainbow trout (29). This gene codes for Blimp1, a typical plasma cell differentiation marker (47). Consequently, as occurs in mammals (47), this *prdm1a* up-regulation went along with a down-regulation of *pax5* transcription. The CCR7^+^ subset showed higher levels of transcription of *ccr7a* but not of *ccr7b*. Even though the anti-CCR7 pAb used in this study recognizes both CCR7A and CCR7B, the fact that only *ccr7a* transcription was up-regulated in IgD^+^IgM^-^ B cells, and especially in sorted IgD^+^IgM^-^CCR7^+^ B cells, strongly suggests that CCR7A is exclusively responsible for the differentiation of the two IgD^+^IgM^-^ B cell subsets. Interestingly, IgD^+^IgM^-^CCR7^+^ B cells also transcribed higher levels of *il1b*. How the secretion of this pro-inflammatory cytokine conditions the role of these IgD^+^IgM^-^ B cells is something to explore in the future. Nonetheless, increased *il1b* transcription was also observed in differentiated rainbow trout blood IgM^+^ B cells when compared to non-differentiated cells (48). Finally, as would be expected from a more differentiated cell, the levels of transcription of secreted IgD were also higher in the CCR7^+^ subset. This increased secretory capacity was further confirmed collecting the supernatants of cultured sorted populations and examining the amount of secreted IgD by ELISA.

In conclusion, we have established that a high proportion of CCR7^+^ cells in the skin correspond to IgD^+^IgM^-^ B cells. Within the IgD^+^IgM^-^ B population, CCR7^+^ cells, which can be found exclusively in the epidermis layer, have a transcriptional profile of cells that are further differentiated to a plasma-like profile, transcribe higher levels of *il1b*, are more complex and secrete more IgD than cells not expressing this chemokine receptor. This is the first report of a differentiation marker for IgD-secreting plasma-like cells, and represents a step forward towards deciphering the role of IgD-switched cells and secreted IgD in mucosal surfaces. How CCR7 expression conditions the homing pattern of these cells and their possible cooperation with other leukocyte subsets, such as with T cells, warrants further investigation.

## Data Availability

The raw data supporting the conclusions of this article will be made available by the authors, without undue reservation.
