# Supplementary material for: CCR7A defines a subpopulation of IgD+IgM- B cells with higher IgD secreting capacity in the rainbow trout skin
Source: Front Immunol. 2025 Feb 14;16:1538234. doi: 10.3389/fimmu.2025.1538234 (PMC11868095; doi:10.3389/fimmu.2025.1538234)
Supplement: Supplementary file 1 [file DataSheet1.pdf]

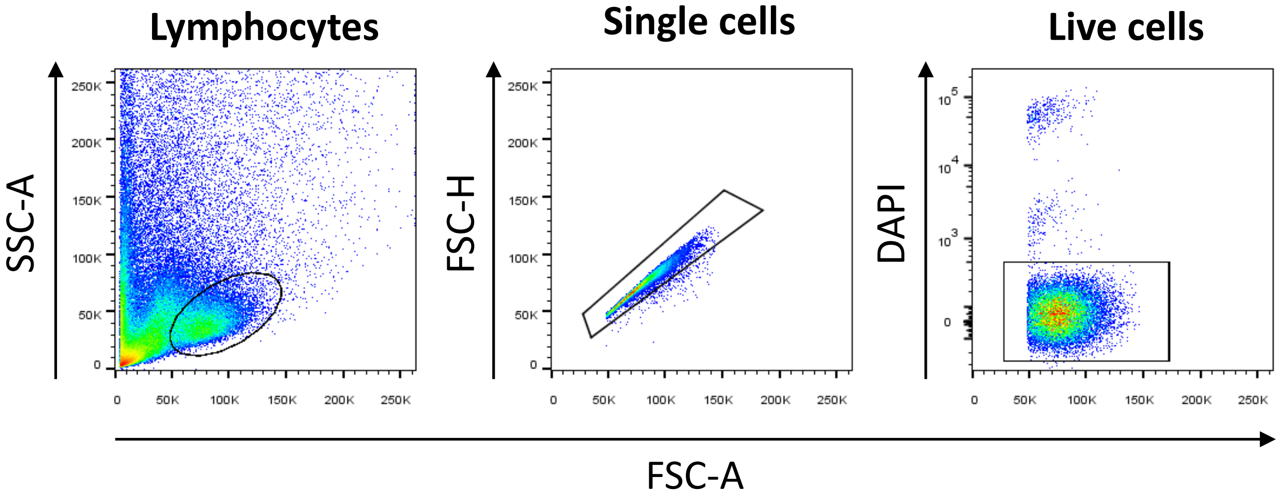

Supplementary Figure 1. Gating strategy. Leukocytes were isolated from trout skin. FSC/SSC profile including a defined gate for lymphoid cells is shown. FSC-H/FSC-A profile within the lymphoid gate indicates singlets. DAPI negative cells within singlet gate were gated in order to select alive cells.

A

B

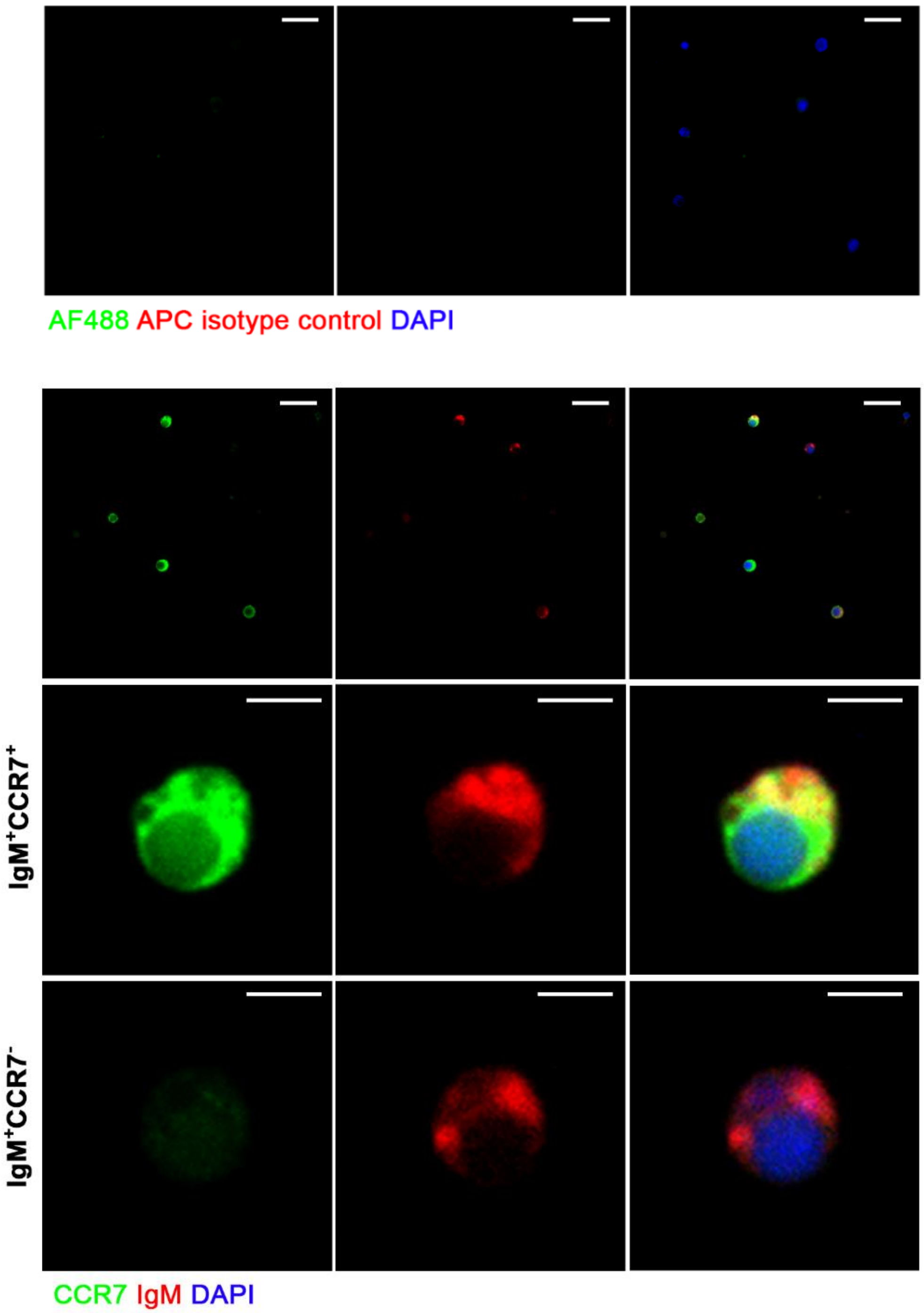

Supplementary Figure 2. Immunofluorescence analysis of skin lymphocytes under the confocal microscope. (A) Images of isotype controls. Scale bars: 20 µm. **(B)** Visualization of skin IgM^{+}IgD^{-}CCR7^{+} and IgM^{+}IgD^{-}CCR7^{-} B cells under the confocal microscope. Examples of each subset are shown. Scale bars: 20 µm uppest images); 5 µm (lowest images).

Supplementary Table 1. List of primers used in this study for real-time PCR analysis of gene expression.

**Gene name**	**Forward primer (5´-3´)**	**Reverse primer (5´-3´)**
b-actin	TCCTTCCTCGGTATGGAGTCT	TTACGGATGTCCACGTCACAC
ccr7a	TTCACTGATTACCCCACAGACAATA	CAGACCGAGGAAGCAGATGAG
ccr7b	AATGGTTACTCCACAGACACGAAC	CAGACCGAGGAAGCAGATGAG
mhc II	ACACCCTTATCTGCCACGTC	TCTGGGGTGAAGCTCAGACT
secreted IgD	TGAACATATCCAAACCAGGTGTCTG	GTCCTGAAGTCATCATTTTGTCTTGA
Il1 b	GACATGGTGCGTTTCCTTTT	ACCGGTTTGGTGTAGTCCTG
prdm1a-1	CAGCGCCCCAGTCAAGATA	GGGGGTAGAGGGCACAGC
prdm1a-2	CATTCGGCCCTATGTGTGG	CCCCTCGGTAGTCAACATGG
prdm1c-1	TCACTGCATCAACACCGAGA	CCGGTCTCCATCACCATCTT
prdm1c-2	CGCCAATGGGAATATGTCA	GACATAGCCAGGATGCAGA
bcma	ATGTCAGAAGGACAGTGTGGACTGG	CGGCTCTGGGGCTTTGCTCT
irf4	CGCATCACCATAGCAACACC	CTCCTCTCCCCAGGCTTTCT
pax5	ACGGAGATCGGATGTTCCTCTG	GATGCCGCGCTGTAGTAGTAC
igd	AGCTACATGGGAGTCAGTCAACT	CTTCGATCCTACCTCCAGTTCCT
